# Tools and approaches to study the human gut virome: from the bench to bioinformatics

**DOI:** 10.1128/msystems.01002-25

**Published:** 2026-02-04

**Authors:** Haley Anne Hallowell, Justin Malogan, Jotham Suez

**Affiliations:** 1 W. Harry Feinstone Department of Molecular Microbiology and Immunology, Johns Hopkins Bloomberg School of Public Health 25802 , Baltimore, Maryland, USA; Technion Israel Institute of Technology, Haifa, Israel

**Keywords:** human microbiome, human virome, bioinformatics, bacteriophages

## Abstract

The human gastrointestinal tract is home to a diverse community of microorganisms from all domains of life, collectively referred to as the gut microbiome. While gut bacteria have been studied extensively in relation to human host health and physiology, other constituents remain underexplored. This includes the gut virome, the collection of bacteriophages, eukaryotic viruses, and other mobile genetic elements present in the intestine. Like gut bacteria, the gut virome has been causatively linked to human health and disease. However, the gut virome is substantially more difficult to characterize, given its high diversity and complexity, as well as multiple challenges related to *in vitro* cultivation and *in silico* detection and annotation. In this mini-review, we describe various methodologies for examining the gut virome using both culture-dependent and culture-independent tools. We highlight *in vitro* and *in vivo* approaches to cultivate viruses and characterize viral-bacterial host dynamics, as well as high-throughput screens to interrogate these relationships. We also outline a general workflow for identifying and characterizing uncultivated viral genomes from fecal metagenomes, along with several key considerations throughout the process. More broadly, we aim to highlight the opportunities to synergize and streamline wet- and dry-lab techniques to robustly and comprehensively interrogate the human gut virome.

## INTRODUCTION

The human gut microbiome is a complex community of bacteria, archaea, fungi, protozoa, and viruses that reside within the gastrointestinal (GI) tract (1). Notably, much of the literature regarding the gut microbiome focuses exclusively on the composition and function of commensal bacteria, although other components, such as resident gut viruses, have been mechanistically linked to mammalian host physiology and disease pathogenesis (2, 3). This collection of viruses, termed the gut virome, is dominated by bacteriophages (or phages) and also includes archaeal viruses, eukaryotic viruses, endogenous retroviruses (ERVs), mycoviruses, and other mobile genetic elements (MGEs), such as plasmids (4, 5).

Alterations in the gut virome have been associated with diverse aspects of human health, including metabolic (6–8), neurocognitive (9), autoimmune (10), and inflammatory diseases (11, 12). Similar to fecal microbiome transplantations (FMT), gut virome transplantations are used by researchers in preclinical models to establish a causal role for the virome in modulating the mammalian host’s health (13–16). Indeed, fecal virome transplantation (FVT) is emerging as a potential therapeutic, showing promise in improving outcomes in models of obesity (15, 16), type 2 diabetes (15), and stress (13), and as a treatment for recurrent *Clostridioides difficile* infections in patients (14). Administration of specific phage consortia in the form of phage therapy can serve not only to selectively antagonize pathogenic bacteria but also as a strategy for targeting pathobionts involved in the pathogenesis of non-communicable diseases. Phage therapy was used in this manner against *Fusobacterium nucleatum,* leading to the inhibition of colorectal cancer cell growth *in vitro* (17). Furthermore, phage therapy targeting *Klebsiella pneumoniae* reduced disease severity in a mouse model of metabolic dysfunction-associated steatotic liver disease (18) and inflammatory bowel disease (IBD) (19) and was safe and well-tolerated when administered to healthy humans (19).

A growing number of studies are deciphering the mechanisms by which endogenous viruses impact homeostasis and disease pathogenesis (20–24). These can be mediated by functional modulation of the microbiome, either through community restructuring (20, 21) or by providing bacteria with new traits (22). In addition, eukaryotic viruses (23, 24), ERVs (25, 26), and even phages, when transplanted to germ-free (GF) mice (2, 27), can modulate health by directly interacting with the mammalian host cells and immune system, triggering both virus-specific and non-specific immune responses.

Historically, research on human-associated viruses primarily focused on disease-causing infectious agents. The advent of human microbiome research has sparked growing interest in the roles commensal viruses play in shaping human host physiology and modulating human health (28, 29). Recently, the United States National Institutes of Health approved the Human Virome Project, a 10-year common fund aimed at characterizing and identifying the human gut virome, as well as developing new tools and methods for this purpose (30). Indeed, computational tools for exploring the gut virome have significantly improved in recent years, enabling the identification of more than 15 million uncultivated viral genomes (UViGs) (31). The improvement of viral protein and sequence databases and computational tools to characterize the virome, combined with standardized reporting (32), has enabled the description of the gut virome in infancy and aging (33–35) as well as multiple disease states (6, 36) in large clinical cohorts. Despite the growing recognition of the relationship between human health and the gut virome, it remains largely underexplored, with many *in silico* findings having yet to be experimentally validated, partially due to challenges in viral cultivation. Experimental validation of these *in silico* observations is critical for translating virome research into clinical applications, and many of the *in vitro* approaches originally developed to study pathogenic viruses have been adapted to explore commensal viruses (37).

**Fig 1:**
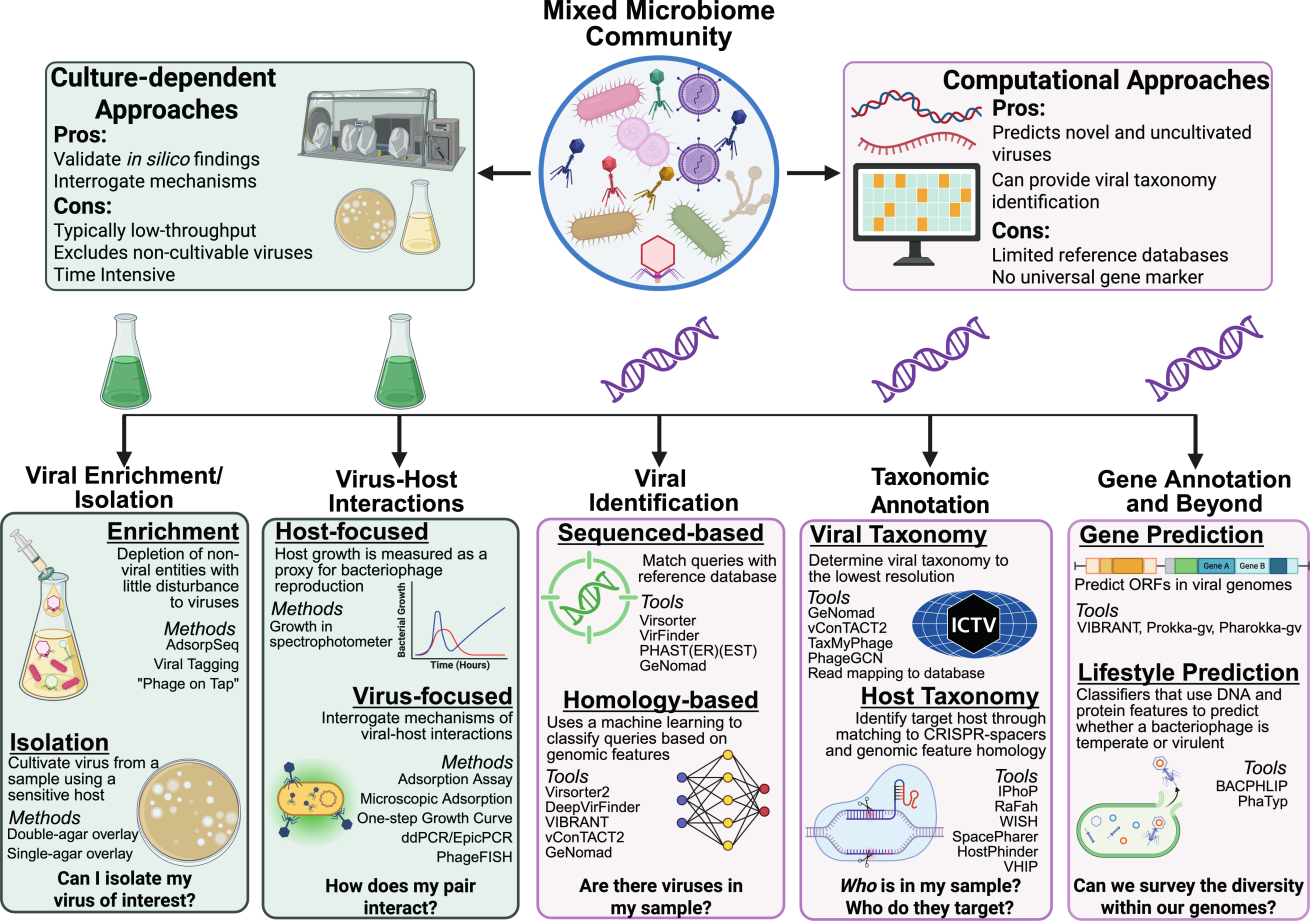
An overview of available culture-dependent and culture-independent approaches to characterize the human gut virome. Both culture-dependent and culture-independent approaches should be used to study the gut virome. At the bench, many protocols have been adapted to enrich viruses in a sample and isolate them using a sensitive host. Once a virus is isolated, various assays can be used to characterize how the virus interacts with its bacterial host. On the command line, viral genomes can be predicted and viral taxonomy can be identified within a sample. Once procured, multiple tools can be used to annotate viral genomes for predicted gene content and viral lifestyle and predict bacterial host taxonomy.

In this review, we describe experimental and computational approaches used to analyze the human gut virome (Fig. 1) and how they have led to important advances in understanding the impact of the gut virome on human health, including evidence for causality in pathogenesis through both direct and indirect mechanisms. Although many of the methods discussed may be broadly applicable to any ecosystem, we will specifically highlight uses of these methods in the gut as well as any gut-specific databases or protocols.

## ENRICHMENT AND ISOLATION

The first step in experimental interrogation of virus-bacterial host interactions is obtaining individual viruses or viral communities of interest. A limited number of gut-associated phages and eukaryotic viruses can be obtained from stock centers such as the American Type Culture Collection, the Leibniz Institute German Collection of Microorganisms and Cell Cultures (DSMZ), the Félix d'Hérelle Reference Center for bacterial viruses of the Université Laval, and the Bacteriophage Bank of Korea, a repository of phages mostly targeting pathogenic bacteria. Laboratory strains of viruses aid research since their genomes are often well-defined, and they can be easily cultivated. In contrast, studying viruses directly cultivated from the gut or other environments enables the exploration of unknown genotypic and phenotypic diversity. Environmental sources of gut-associated commensal viruses include wastewater and fecal samples (38, 39). Many of the methods discussed below were originally developed to enrich and cultivate phages from environmental reservoirs such as oceans (40) or soils (41, 42) but have since been co-opted for the enrichment and isolation of gut-associated viruses.

### Enrichment of viruses from environmental samples

The goal of viral enrichment is to deplete a sample of non-viral entities while causing minimal disturbance to viral particles. Viral enrichments can then be utilized to isolate viruses in culture or for genomic sequencing. There are multiple strategies that can be used to enrich a sample depending on the specific aim of the experiment. Some enrichment strategies aim to separate viruses from environmental samples using size-based exclusion or chemical-based procedures. These techniques, along with their impact on downstream applications such as metagenomic sequencing, are discussed below.

To enrich commensal viruses from environmental samples, various protocols have been successfully adapted from those used in pathogenic virus research (43, 44). These protocols include the use of size- and chemical-based methods to deplete samples of other microorganisms and debris. Size-based methods utilize filters with specific pore sizes to physically separate viruses from larger particles (16, 44, 45). Meanwhile, chemical-based separation involves precipitating viruses from a solution (46) or lysing membrane-bound microbes using chloroform or lysozyme (11, 47, 48). When the goal of the enrichment is sequencing, DNase/RNase treatments are typically applied before nucleic acid extraction and viral lysis to deplete the sample of nucleic acids from environmental sources or from non-viral microorganisms (46, 47). Typically, these techniques are combined to improve the quantity and quality of viral enrichments (49).

These methodologies have been evaluated for their efficiency and reproducibility across multiple studies (50, 51). Importantly, all enrichment methods have been found to display bias toward or against certain groups of viruses (46, 48, 51–54). Although fast and efficient, viral recovery from size-based separation methods can physically damage virions and be biased against viruses larger than the filter pore size, and the type of filter can impact which virions are recovered (46, 51, 52). Chloroform is commonly used to lyse bacteria in a sample during chemical-based separation, which can improve yield by releasing intracellular virions (53), but it also disrupts the membranes of enveloped viruses (48, 54). Thus, it is crucial to consider the physical characteristics of potential virions of interest when selecting an enrichment strategy. Notably, there is growing evidence that, as with the gut bacteriome (55, 56), the diversity and composition of the gut virome vary along the mammalian GI tract (57, 58) as well as between luminal and mucosal samples of the same GI region (57, 59), a variation that is not fully captured by fecal samples. To this end, methods for enriching viruses from different fractions, such as the mucosa, are being improved. An enrichment method to liberate and enrich viruses from the gut mucosa recently demonstrated its effectiveness with samples from the domestic pig (*Sus scrofa*) and rhesus macaque (*Macaca mullata*) (57). Similar methods have been used to describe differences in the ileal and colon mucosal virome in Crohn’s disease and ulcerative colitis, respectively (12, 60).

### Viral isolation

Once the viruses are enriched from a sample, phage-sensitive bacterial hosts can be used to isolate viruses of interest. While enrichment aims to separate viruses from cellular components, viral isolation aims to cultivate a virus of interest from a complex community of viruses. Phages are typically incubated with sensitive hosts to increase the quantity of virions and then plated on an agar plate using the double-agar overlay method, the primary isolation method (61). Plaques on the agar plate represent a single phage and its progeny, which infect and lyse sensitive bacterial hosts, leading to clearing of the bacterial lawn. Individual plaques can then be picked from the agar plate and further processed to produce a pure phage monoculture (61).

However, not all phages can be isolated using the double-agar overlay method. Viral tagging (VT) represents an alternative isolation method that has already been successfully applied to fecal samples (62, 63), does not rely on plaque formation, and can identify bacterial hosts (discussed further in “*In vitro* approaches to bacterial host range identification”). In this method, viromes enriched from an environmental sample are labeled with a fluorescent dye that non-specifically binds to nucleic acids. Once labeled, viruses are incubated with one or more bacterial hosts. After incubation, bacterial cells infected with (or bound by) labeled phages are sorted from the culture using fluorescence-activated cell sorting and undergo sequencing. This method was initially shown to be successful using marine samples and associated cyanobacteria (64). When this method was adapted for use with gut samples, it enabled the characterization of over 350 unique virus-bacterium pairs from healthy human volunteers (62). More recently, VT was performed on fecal samples from patients with IBD and healthy matched controls to isolate novel rare phages against *Fecalibacterium prauznitzii* that were originally not detected with typical metagenomic sequencing of whole fecal samples or fecal viromes (63). AdsorpSeq utilizes a similar method to isolate phages from enriched viromes and identify bacterial hosts (65). Here, viromes are incubated with cell envelopes isolated from cultivated bacterial hosts of interest, rather than cultivated or uncultivated whole bacteria, as done in VT. Adsorbed and unadsorbed phages are separated using gel electrophoresis. Phages adsorbed to a cell envelope can then undergo genomic sequencing for identification. AdsorpSeq was able to isolate phages from wastewater samples (65), demonstrating its applicability for studying gut-derived phages. The reliance on cell envelopes likely makes AdsorpSeq more suitable for identifying gram-negative hosts whose outer membranes contain phage receptors and are more readily isolated. However, VT requires fluorescent labeling of hosts, which may render it less suitable for high-throughput screens and can be technically challenging in some bacteria.

While VT and AdsorpSeq are effective at isolating phage and identifying novel phage-bacterium pairs, they cannot distinguish viable from non-viable phages and assume that adsorption always leads to successful infection (65). A variation of VT called “viral tag and grow” aims to address this limitation by adding an incubation step to determine whether viable phages are produced (66). While this method has been applied to *Escherichia coli* and its respective phages (67), its applicability to other human gut bacteria-phage pairs remains to be determined. A limitation for all methods that incubate phages with bacteria or bacterial envelopes is that these interactions may not manifest similarly in the GI tract. The difference between artificial laboratory growth conditions and those naturally found in the gut can alter gene expression in the bacterial host, which, in turn, may affect the capacity of gut-derived phages to bind (68).

### Impact of viral enrichment on sequencing

Viral enrichment of fecal samples prior to sequencing can significantly impact the recovery of viral DNA. For example, protocols developed with an emphasis on DNA viruses significantly reduce the recovery and characterization of RNA viruses (69). Nucleic acid yield can be low from virus-enriched samples. Thus, nonspecific nucleic acid amplification via multiple displacement amplification (MDA) (50) has been widely used prior to the sequencing of enriched samples. However, MDA is biased by the abundance of the template in the sample and its accessibility for priming, including factors like GC content, fragment size, and DNA structure (e.g., biased toward circularized DNA) (50).

Bulk metagenomic sequencing also biases the recovered viruses toward temperate or actively infecting viruses, thereby underrepresenting the free viral fraction and viruses not actively infecting their bacterial host (70). Additionally, the gut microbiome contains many low-abundance members that, despite their low abundance, can play a disproportionately large role in the gut ecosystem (71). Non-omics methods, such as VT, can facilitate the detection of viruses that target these members. While traditional sequencing methods recovered a few lysogenic phages targeting *Fecalibacterium prauznitzii* (72, 73), a functionally important commensal consistently depleted in patients with IBD, VT enabled the recovery of multiple previously undetected *F. prauznitzii*-targeting phages (63). Conversely, viruses targeting highly abundant bacteria, particularly prophages, are overrepresented in metagenomes, whereas viruses targeting rarer bacterial populations may be underrepresented or absent (74).

An ongoing discussion in the field asks whether the depletion of non-viral microorganisms in fecal samples increases the yield and quality of recovered viral genomes compared to traditional bulk metagenomic sequencing (69, 75, 76). Overall, few studies have directly investigated whether viral enrichment improves viral recovery. A recent report comparing paired metagenomes and viromes found that enrichment only slightly improved viral genome recovery, in line with previous literature (75, 76). Although the number of viral genomes did not differ between strategies, enrichment led to a significant increase in viral read mapping rates and the overall coverage of some viral genomes. This trend was not observed across all environments, as viral recovery was significantly improved in soil samples, as previously documented (75, 77). Additionally, metagenomes and viromes were each found to contain unique viral genomes not recovered in the other, indicating that either approach alone is not inclusive of all gut viral diversity (75). In light of this, when investigating the gut virome, it is most advisable to sequence paired bulk and virus-enriched fecal samples. This sequencing strategy can capture the greatest viral diversity within the fecal microbiome while still allowing the characterization of virus-bacteria dynamics and identification of bacterial hosts. If this strategy is not feasible, the biological question should drive the decision to enrich a sample. Enrichment would be beneficial in cases where the virus of interest is rare or not actively replicating in bacterial cells, as with non-bacterium-targeting viruses. If viral-host interactions are being examined in an environment, bulk sequencing would be required to capture bacterial host abundances adequately.

## GENOME SEQUENCING AND CLASSIFICATION

While *in vitro* methods can identify and quantify different steps in virus-host interactions, including infectivity rate, host range, and progeny production, only a small fraction of the global virome has been cultivated. Utilizing culture-independent methods, such as shotgun metagenomic sequencing, in combination with computational tools (Table 1), to predict and characterize viral genomes more accurately captures viral diversity within a sample (78). Notably, predicting virome composition is more challenging than predicting bacterial composition. This is in part due to current sequence databases only capturing a portion of the estimated global viral diversity and because there is no universal viral marker gene, such as the *16S rRNA* gene in bacteria (79).

**TABLE 1:** Computational programs for the identification, annotation, and prediction of viral genomes

Tool (reference)	Approach	Accessibility	Similar tool(s)
MetaviralSPAdes (80)	*De novo* short read assembler Modified Metaspades tool to better suit viral genomes. Can also determine completeness of viral genomes	https://github.com/ablab/spades/tree/metaviral_publication https://github.com/ablab/viralVerify/	
viralFlye (81)	*De novo* long-read assembler. Modified Flye tool to better suit viral genomes	https://github.com/Dmitry-Antipov/viralFlye	
CheckV (82)	Assess the quality of UViGs using a large marker database and removes host contamination	https://bitbucket.org/berkeleylab/CheckV https://github.com/ablab/viralComplete/	ViralComplete (80)
DeepVirFinder (83)	Uses a convolutional neural network to predict if a query contig is a virus. Trained on viruses on RefSEQ from May 2015 and before	https://github.com/jessieren/DeepVirFinder	
GeNomad (84)	Hybrid approach: Uses both a reference-based and deep neural network to predict if a query contig is a virus. Can predict viruses of all genome types and plasmids. Can taxonomically annotate UViGs. Trained on ~200k proteins from predicted MGEs.	https://portal.nersc.gov/genomad	
PHASTEST (85)	Identify viral contigs using a reference database	https://phaster.ca/	PHAST (86), PHASTER (87), ViralVerify (80)
PPR-Meta (88)	Uses a convolutional neural network to predict if a query contig is a virus. Can predict other mobile genetic elements. Trained on all viruses and plasmids from NCBI (published 2019).	https://github.com/zhenchengfang/PPR-Meta	PhaGCN (89)
VIBRANT (90)	Uses a supervised neural network to classify query contigs as viral using amino acid sequence homology. Can predict other mobile genetic elements whether a query sequence is a plasmid. Provides functional annotation of genes, including auxiliary metabolic genes	https://github.com/AnantharamanLab/VIBRANT	
Virsorter (91)	Identify viral contigs using a reference database	https://github.com/simroux/VirSorter.git	
Virsorter2 (92)	Trained random forest classifiers predict viral contigs. Trained on viruses on RefSEQ from 2020 and before, as well as custom UViGs from various environments	https://github.com/jiarong/VirSorter2	
VIRIDIC (93)	Calculates the intergenomic distance between viral genomes.	https://github.com/CristinaMoraru/VIRIDIC	
TaxMyPhage (94)	Aligns kmers to a reference database to determine taxonomy.	https://github.com/amillard/tax_myPHAGE	
VICTOR (95)	Infers taxonomy using phylogenetic and clustering methodologies.	https://victor.dsmz.de	
vConTACT2 (96)	Clusters query UViGs with reference genomes using a gene-sharing network to infer taxonomy and identify viruses.	https://bitbucket.org/MAVERICLab/vcontact2	
HostPhinder (97)	Compares kmers to a database of phages with known hosts.	https://github.com/julvi/HostPhinder	
IPHoP (98)	Pipeline that determines target host taxonomy by using six host prediction tools and developed rulesets to calculate the most robust host annotation for each UViG.	https://bitbucket.org/srouxjgi/iphop/src/main/	
RaFaH (99)	Random forest classifier to predict target host. Trained on ~44k protein clusters from diverse environments.	https://sourceforge.net/projects/rafah/	
SpacePharer (100)	Aligns UViGs to a reference database containing CRISPR spacers from metagenomic data.	https://github.com/soedinglab/spacepharer	
Virus-Host Interactions Predictor (VHIP) (101)	Resolves a complex network of user-provided phage and bacterial genomes to predict multiple bacterial hosts.	https://github.com/DuhaimeLab/ VirusHostInteractionPredictor	VirHostMatcher (102)
Who is the host? (WISH) (103)	Alignment-free, compares frequencies of kmers between UViGs and bacterial genomes.	https://github.com/soedinglab/WIsH	
BACPHLIP (104)	A random forest classifier to predict UViG lifestyle (eg, temperate, virulent). Trained on 634 phage genomes.	https://github.com/adamhockenberry/bacphlip	PHACTS (105)
PhaTYP (106)	Uses a sequence-based language model to predict UViG lifestyle. Trained on the phage proteins in the RefSEQ Virus database.	https://github.com/KennthShang/PhaTYP	DeePhage (107)
Pharokka (108)	Predicts open reading frames and then annotates them using the PHROGS database.	https://github.com/gbouras13/pharokka	

To taxonomically identify viruses in a sample (whether a mixed community or a homogenous monoculture), genomic sequencing is needed. When a sequenced sample contains multiple viral genomes (as in environmental or fecal samples), the sequencing reads, or short DNA fragments, are aligned to a database of viral genomes. The choice of database to align to, either a curated published database (assembly-free approach) or assembled viral genomes from the sample of interest (assembly-based approach), can significantly impact the final predicted community composition and function (109). Assembly-free methods for gut virome profiling utilize curated viral databases that range from comprehensive repositories, such as IMG/VR (31), to environment-specific collections, including the Metagenomic Gut Virus (MGV) database (109) and the Unified Human Gut Virome database (110), among others (Table 2). However, utilizing these databases assumes that all viruses in the sample of interest are represented within the curated database. However, it has been estimated that up to 90% of viral sequences share little to no similarity with reference databases (111). To overcome these limitations, assembly-based methods are widely used (74), in which viral genomes are reconstructed from samples of interest and used as a reference database. These viral genomes can be fully characterized, including annotation of viral taxonomy and genes, as well as prediction of target bacterial host taxonomy and lifestyle. However, assembly-based methods often yield incomplete genomes and can miss rare viruses in a sample (75). While assembly-free methods alone are useful for surveying metagenomes with shallow sequencing depth or very short read lengths (70), they can also be combined with assembly-based methods to survey metagenomes more comprehensively. For example, this approach has recently been used to explore longitudinal changes in gut virome and plasmid composition in infants born via cesarean section (35). Below, we will briefly describe the steps involved in assembly-based characterization.

**TABLE 2:** Gut-Specific viral databases

Database (reference)	Description	Accessibility
Gut Phage Database (GPD) (112)	Derived from 28,000+ human gut samples and 2,898 culturable bacterial reference genomes. Samples from 28 different countries. Samples from six continents. ~142,000 non-redundant phage genomes.	http://ftp.ebi.ac.uk/pub/databases/metagenomics/genome_sets/gut_phage_database/
Human Virome Database (HuVirDB) (113)	Phage genomes based on protospacer identification via CRISPR system.	github.com/jbisanz/HuVirDB
Metagenomic Gut Virus (MGV) (109)	189,680 viral genomes from human fecal samples. DNA-virus genomes. Viruses specifically from human samples.	https://portal.nersc.gov/MGV
Unified Human Gut Virome (UHGV) (114)	Comprehensive collection of viral genomes from 12 other viral databases: Metagenomic Gut Virus Compendium (MGV) Gut Phage Database (GPD) Metagenomic Mobile Genetic Elements Database (mMGE) IMG Virus Resource v4 (IMG/VR) Hadza Hunter-Gatherer Phage Catalog (Hadza) Cenote Human Virome Database (CHVD) Human Virome Database (HuVirDB) Gut Virome Database (GVD) Atlas of Infant Gut DNA Virus Diversity (COPSAC) Circular Gut Phages from NCBI Danish Enteric Virome Catalogue (DEVoC) Stability of the human gut virome and effect of gluten-free diet (GFD)	https://github.com/snayfach/UHGV
PHROG (115)	A database of viral proteins consisting of ~40k viral protein clusters.	https://phrogs.lmge.uca.fr/
VOGdb (116)	Viral Orthologous Group protein database generated from and updated with RefSEQ.	https://vogdb.org/

### Viral genome classification

Before assembly-based characterization can occur, viral genomes need to be assembled and predicted from the sequencing data. In general, when analyzing metagenomes, low-quality reads and reads from the mammalian host should be removed before any workflows. In some cases, bacterial reads can be depleted, but this may also remove prophage sequences that contain bacterial genes. Computational depletion of bacterial sequences is less important for virus-enriched samples, as bacterial cells are removed before sequencing. Once filtered, the reads are assembled into contigs. The choice of metagenomic assembler may affect the number and quality of recovered viral contigs, with some programs, such as MetaSpades (117) and MEGAHIT (118), performing better in terms of completeness and length of viral contigs (119). There is a slight trade-off between accuracy and the length of the final contig in some assemblers, such as Velvet or ABySS (119). Virus-specific assemblers such as metaviralSPAdes (80) and ViralFlye (81) have been published for short-read and long-read sequencing data sets, respectively. However, no independent benchmarking has been conducted to determine whether these are superior to popular metagenomic assemblers, such as MEGAHIT (118) and MetaSpades (117).

After assembly, contigs are classified as bacterial or viral, which yields viral genomes for downstream analyses and depletes bacterial sequences from a sample. To do this, viral prediction programs use various strategies, including aligning against reference databases of viral sequences or using machine learning models to classify contigs as viral. Programs that aim to align user query sequences to a reference database include Virsorter (91), PHAST (86), PHASTER (87), and PHASTEST (85). Alternatively, tools such as VirFinder (120) and DeepVirFinder (83), PPR-Meta (88), Virsorter2 (92), and VIBRANT (90) utilize a different approach that does not require a database and can classify viral sequences *de novo*. In particular, they utilize machine learning methods as a means to identify viral signatures important for detection, as well as greatly expanding reference marker databases for reference-dependent classifiers (121). When compared across soil, ocean, and human gut biomes, programs that used machine learning were more effective at classifying viral contigs than those that relied solely on a reference database (122, 123). Benchmarking of GeNomad, which uses both a reference-based and an unsupervised machine learning classifier, showed that it outperforms single-strategy tools in recovery and identification (84). When using tools that utilize machine learning, it is important to understand which training data were used and the similarity between the test and training data. For example, in some benchmarking, Virsorter2 performed similarly to other machine-learning-based tools with ocean and soil biomes but outperformed many others with gut biome samples. The authors postulated that this was due to the inclusion of gut virus genomes into the training data set of Virsorter2 (122).

Once classified, UViGs can be assessed for completeness and contamination using tools such as ViralComplete (80) and CheckV (82). The most widely used tool for this purpose is CheckV, which, in addition to estimating the completeness and contamination of each genome, removes bacterial host contamination by aligning small segments of the sequence to a database of known bacterial hosts (82). Each contig is assessed and assigned a completeness score aligning with published standards (74). CheckV is commonly used to assess UViG quality and contamination in the gut virome. CheckV was used to assess viral genomes before constructing high-quality, gut-specific viral databases, including the MGV (109), UHGV (114), Gut Virome Database (76), Human Virome Database (113), and the Early-Life Gut Virome catalog (34). Beyond public database construction, CheckV has been used in numerous studies of the gut virome, including those investigating genome topology (124), IBD (125), and metabolic syndrome (6).

It is likely that one or more recovered genomes will be redundant. Because of this, it is best practice to dereplicate recovered viral genomes (i.e., remove redundant genomes from the UViG collection) to accurately quantify the viral community within the sample and increase computational efficiency. Although dereplication is extremely common in viromic analyses, clustering viral genomes inherently masks some microdiversity within viral populations. Programs such as MMseqs2 (126), CD-HIT (127), or the virus-specific tool VIRIDIC (93) calculate average amino acid or nucleotide identity between viral genomes and cluster them. The cluster representative, typically the longest and most complete genome, is then used to assign characteristics such as taxonomy or lifestyle.

Importantly, a large fraction of viral contigs recovered from metagenomes will not be complete (128), potentially hampering the detection of viral lifestyle and metabolic capabilities. Binning, which groups contigs into a consensus genome based on sequence homology, can resolve fragmented genomes. However, binning involves a trade-off between maximizing completeness and masking true diversity within samples. Binning of viral genomes has proven more difficult than traditional binning strategies for bacterial or archaeal genomes, due, for example, to the lack of universal viral marker genes to help delineate genome bins. vRhyme was specifically developed to address this issue during viral genome binning (129). Using a supervised machine learning approach, vRhyme significantly improved the completeness of binned UViG genomes while reducing bin contamination, as demonstrated by its own benchmarking (129).

Some viral genomes are at too low abundance to be detected in metagenomic samples. In the case of low-abundance prophages, single-cell sequenced bacterial genomes, termed single amplified genomes (SAGs) (130), can be used to recover these genomes. In contrast to binned bacterial metagenome-assembled genomes, which can represent a consensus sequence and lack mobile genetic elements (131), SAGs retain their heterogeneity and MGEs. This method also enables the direct identification of bacterial hosts. Indeed, bacterial SAGs from diverse environments, including the ocean and human mouth, skin, and gut biomes, have yielded numerous novel viral genomes and microdiversity (63, 132–135). In the gut, novel phages not detected by traditional viromics pipelines have been recovered from SAGs (63) and include viruses targeting *F. prauznitzii* (63), *Bifidobacterium* spp., and *Ruminococcaceae* spp. (135).

### Assigning viral taxonomy

After a contig is predicted to be viral, computational tools can assign viral taxonomy, and many characteristics of the virus can be annotated. Assigning taxonomy to viral genomes can prove difficult, given the challenges discussed above, including limited reference databases, incomplete assembled UViGs, and a lack of a universal gene marker. This is made more complex as viral taxonomy is currently being modernized, with the major, non-monophyletic viral families based on morphology (myo-, podo-, and siphoviridae) being recently abolished by the International Committee on Taxonomy of Viruses (ICTV) in 2021 (136). To overcome these technical challenges, various strategies have been developed to classify UViGs taxonomically. For example, predicted genes can be aligned to a large marker database, as is the case with GeNomad (84). Alternatively, programs may use clustering algorithms to group user query genomes. vConTACT2 (96) and VICTOR (95) cluster query viral genomes based on shared gene sequences and situate them in a gene-sharing network or phylogenetic tree, respectively. Taxonomy can be inferred from reference genomes, which are typically included in clustering (96). Reference genomes can be used to train a machine learning model to predict the taxonomic classification of UViGs based on genomic features, as is the case with PhageGCN (89), which also continually updates to incorporate the latest ICTV taxonomy. Finally, TaxMyPhage uses short unique fragments of each phage genome, called kmers, and compares them to a database of known phage genomes. Importantly, it assumes that the viral genome is complete; thus, caution should be taken when using UViGs (94).

## BACTERIAL HOST RANGE PREDICTION AND IDENTIFICATION

Determining the bacterial host range of viruses in a sample is often a highly valuable step in viromics analyses. The gut virome can impact host health by modulating the gut microbiome (137). Therefore, characterizing bacterial hosts that phages of interest predate can provide further context for phage-mammalian host health relationships. *In silico*, multiple strategies are used to predict which bacterial host a given phage may target. However, prediction *in silico* remains difficult, as tools can only reliably predict bacterial hosts at the genus level or higher (138). To complement these *in silico* techniques, multiple *in vitro* methods can readily identify bacterial hosts of interest, some of which do not require culturing the host. Below, we will discuss these *in silico* and *in vitro* approaches to predict and identify virus-bacterial host ranges.

### *In silico* approaches for predicting phage-bacterial host ranges

Because of technical challenges in bacterial and viral cultivation, it is not practical to determine bacterial host range solely *in vitro. In silico*, most bacterial host prediction tools attempt to search for bacterial signals in viral genomes or vice versa. This can be achieved by aligning viral sequences with large databases of CRISPR spacers, a strategy used by programs such as SpacePharer (100). However, not all bacteria have CRISPR spacers, and CRISPR arrays are limited by their size to recently infecting phages, which further complicates this method (138). Bacterial hosts can also be predicted through alignment-free methods, where, for example, kmers shared between bacterial and viral genomes are counted, as with WiSH (103). In contrast, other tools attempt to associate UViGs with phages that have known bacterial hosts, known as “guilt by association.” These tools will align UViGs to databases of phages with known bacterial hosts (HostPhinder) (97) or use those databases to train machine learning models to predict bacterial hosts (RaFaH) (99). Recently, a workflow integrating many of these tools, iPHoP, was introduced. iPHoP employs a standardized rule set to determine the most robust annotation from one of six tools for each viral genome (98). Finally, unlike the programs discussed above, some tools, such as VirHostMatcher (102) and Virus-Host Interactions Predictor (VHIP) (89), utilize networks to predict phage-bacterial host pairs. Virus-Host Interactions Predictor (VHIP) can resolve a complex network of query phages and bacterial genomes reconstructed from metagenomes at ~88% accuracy to predict multiple bacterial hosts for each UViG rather than only the top annotation (101). Beyond these bacterial host prediction tools, correlations between bacteria and phage abundances have been used to both predict bacterial hosts (139) and validate bacterial host prediction tools. In the gut, this method has been used to validate predicted bacterial hosts of viruses in the Gut Phage Database and found that the predicted bacterial host and phage co-occurred 92% of the time (112).

Proximity-based methods have also been used to link phages with their target bacterial hosts. In addition to the VT discussed above, chromosomal confirmation capture (HiC, proximity ligation, or meta3C) is an approach that overcomes the limitation of PCR-based assays, which require prior knowledge of the phage and bacterial genomes to generate markers/probes. This method uses formaldehyde to cross-link DNA to other nearby DNA molecules within a cell (140, 141). The bacteria-phage hybrid DNA structures can then be computationally analyzed with ViralCC (142), a specialized program for annotating hybrid DNA structures from HiC, to identify which phage was inside a target bacterial cell at the time of treatment. HiC has been used successfully to identify novel putative phage-bacterium interactions in both murine (140) and human fecal samples (141). The HiC and ViralCC workflow represents the synergy between *in vitro* and *in silico* approaches for determining phage-bacterial host pairings.

### *In vitro* approaches to bacterial host range identification

Many approaches used to isolate a specific phage from a complex community can also identify the phage’s bacterial host range. For example, VT was initially developed as a high-throughput method to screen various marine phages and determine their bacterial host ranges (143). Similarly, AdsorpSeq was also developed as a method to detect phage-bacterium host pairs (65). AdsorpSeq and VT can provide information on phage-bacterium pairs because they rely on phage adsorption to a specific bacterial host (65, 143). However, the results may be misleading when using gut commensals *in vitro,* as numerous studies have reported that gut-associated bacteria exhibit different gene expression patterns in the gut environment versus in a laboratory setting (68, 144, 145). As a result, *in vitro* treatment of these bacteria can alter the expression of membrane-associated molecules that phages can bind. This means that these techniques could be excluding phages that would normally bind to an expected bacterial host in the gut but not *in vitro* due to the lack of bacterial ligand expression. The inverse is also possible, in which a phage binds to a bacterial ligand expressed *in vitro* but not in the gut.

To overcome potential issues with culture-based approaches affecting phage-bacterial interactions, a few PCR-based approaches can be used to identify phage-host pairs directly from the sample, thereby bypassing the need for cultures. Digital droplet PCR (ddPCR) is a technique that can be used to identify phage-host pairs (146). This method enables multiplexing of multiple genes within a sample, enabling more accurate detection of low-abundance genes than traditional qPCR (147, 148). Due to these features, ddPCR has been used to investigate phage-bacterial interactions, particularly to quantify phage/bacterial densities and characterize phage competition within the same host (146). Emulsion-paired isolation-concatenation PCR (epicPCR) has been used to identify phages and characterize phage-bacterial host dynamics in a lake estuary (149). In this approach, a primer for the *16S rRNA* gene of the sensitive bacterial host is fused to a phage gene, such as the ribonucleotide reductase gene, and encapsulated within lipid droplets, allowing single-cell resolution (149). While epicPCR has not been applied to the gut microbiome, its success in environmental samples (149) highlights its ability to provide insight into the complex ecology of the gut virome. However, for PCR-based assays to work adequately, the genome of the virus of interest must be known for primer design (146).

## VIRAL QUANTIFICATION

Viral quantification is a critical step in most wet lab and dry lab pipelines. Within *in vitro* approaches, viral quantification, or viral titer, is important for determining the amount of viable virus and critical for the multiplicity of infection calculations. Within *in silico* approaches, viral quantification is necessary to obtain the absolute abundance of viruses within a sample.

### *In vitro* viral quantification

The double-agar overlay method, the primary technique used for isolating phage-bacterium host pairs, is also a method for determining the concentration of viruses in a sample or the viral titer. Once isolated, viral stocks can be serially diluted and plated with their sensitive bacterial host (61). Plaques are then counted, and the viral titer is calculated and reported as plaque-forming units (PFU/mL). While effective, the double-agar overlay method can be time- and reagent-consuming. Thus, a variation called a “single-agar overlay” (150) has been developed, enabling high-throughput phage quantification. The single-agar overlay uses only a single layer of agar, instead of the traditional double layer (150), thereby reducing experimentation time and reagent use. Using gram-negative bacterial pathogen-phage pairs, this method produced visible, countable plaques in 4–6 h compared to 18 h with traditional double-agar overlay methods (150).

It should be emphasized that culture-dependent methods, such as plaque assays, are best-suited for viruses that lyse their target prokaryotic or eukaryotic host and are not useful for viruses that induce no lysis *in vitro*, such as the highly abundant, gut-associated crAssphages (151), ERVs (152), or phages for which the bacterial hosts cannot be identified or cultivated (153). In addition, as previously stated, culture conditions are not representative of the gut environment, which can result in alterations in bacterial gene expression and phage replication that might not naturally occur in the gut (68). In such cases, alternative methods of viral quantification can be used to complement culture-dependent methods (e.g., microscopy-based) (154). Typically, viral quantification by microscopy is performed using fluorescent microscopy, in which virions are tagged with fluorescent dyes and then enumerated (13, 155, 156). Meanwhile, scanning or transmission electron microscopy can be used for enumeration but is better suited for visualization of virion structure due to their higher resolution (154). Unlike plaque-based methods of viral quantification, the major limitation of microscopy-based techniques is their inability to distinguish between viable and non-viable virions (154).

Many eukaryotic viruses can be quantified on sensitive cell lines in plaque-based assays (157). However, a subset of eukaryotic viruses—ERVs—remains difficult to quantify using plaque- or microscopy-based methods. Endogenous retroviruses are genomic sequences that result from ancient retroviruses integrating their genomes into an evolutionary ancestor (158, 159). Over evolutionary history, those ancient integrated genomes accumulated mutations that altered the provirus into what is classified as an ERV (158). As a result of these accumulated mutations, most ERVs do not produce virions that can be quantified via plaque-based methods or microscopy (160). Instead, quantification of ERVs is attributed to expression activity. Such measurements rely on quantifying either ERV mRNA transcripts using RT-qPCR (161) or single-cell RNA-seq (152).

Quantification of so-called “polonies” is another culture-independent, PCR-based method for quantifying groups of DNA viruses within a sample (162). This method uses a set of degenerate primers to quantify viruses from an entire viral genus rather than a single viral species. PCR-based methods can struggle to accurately quantify samples where degenerate primer sets are used, as each probe may amplify targets at varying efficiencies. To overcome this, the polony method physically separates the amplification of each viral DNA template in a polyacrylamide gel (162). Amplification results in “polonies,” or PCR colonies (162), which are then counted as though they were colonies through the use of fluorescent probes. To date, polonies have only been used to quantify viruses from aquatic samples (162); however, this culture-free approach has the potential to be useful for quantifying groups of DNA gut viruses with unculturable bacterial hosts.

Many of the methods above have been used to quantify and characterize various gut phages and phage cocktails targeting gut bacteria. In addition to quantifying viruses in virus-enriched fecal samples, Sutcliffe et al. used epifluorescence microscopy to quantify virus-like particles before and after the administration of various oral medications *in vivo* (153) to measure the impact of these compounds on phage production in the gut microbiome. As mentioned above, electron microscopy is optimal for visualizing viral particle structure, and the structure of ΦCrAss001 phage was resolved using cryogenic transmission electron microscopy (163). Furthermore, using a combination of microscopy, genomics, and mass spectrometry, Shkoporov et al. were able to identify multiple novel viral protein structures and assign some functional roles (163). Microscopy and genomic techniques were also used to recently describe the diversity of ΦCrAss001 phages worldwide (151).

Phage quantification and characterization are critical steps in preparing phage therapy to ensure the safety and efficacy of the treatment (164, 165). Phage therapy is primarily studied against ESKAPE pathogens (43) and has shown efficacy in treating these antibiotic-resistant bacteria, especially when administered concomitantly with antibiotics (166, 167). It has also been applied as personalized phage therapy in 100 cases of treatment-resistant infections (168). Targeting ESKAPE pathogens with their cognate phages is facilitated by ease of cultivation, which is not the case for some commensals. However, an emerging paradigm holds that phage therapy can be used to target endogenous bacteria that, although not causing a prototypical infection, are involved in the pathogenesis of non-communicable diseases. For example, high-alcohol-producing strains of *K. pneumoniae* are associated with an increased risk of non-alcoholic fatty liver disease (169–171). Gan et al. isolated a novel phage, phiW14, against a high-alcohol-producing *strain of K. pneumoniae,* resulting in a marked decrease in observed fat accumulation in murine models (18). These researchers used double-agar overlays to quantify phage titer and transmission electron microscopy to characterize phiW14 (18). Through the use of culture-dependent and -independent methods, a phage cocktail was generated against *K. pneumoniae* strains from the Kp2 clade that were found to be associated with IBD in clinical cohorts (19). Administering the *K. pneumoniae*-targeting phage cocktail suppressed intestinal inflammation *in vivo* (19). When this cocktail was administered to healthy human volunteers, it was safe and well-tolerated (19), providing further support for the use of phage therapy to ablate disease-promoting gut commensals.

### *In silico* viral quantification

In order to detect differences in viral composition and diversity between two samples, it is standard to quantify predicted viral genomes *in silico*. Methods like read mapping quantify the number of reads belonging to viral genomes, which can be further normalized to produce relative abundances of each genome in a given sample. While relative abundances can be used to calculate differences between samples, they cannot be used to calculate total phage loads. It is possible to obtain a semi-quantitative measurement of phage load in a sample by spiking it with a known concentration of a viral standard. To this end, the Lactococcal phage Q33 has been used to quantify the total phage load in fecal samples from healthy human subjects (46, 172) and phage load in mucosal tissues from domestic pig (*Sus scrofa*) and rhesus macaque (*Macaca mullata*) (57). In both cases, phage Q33 was added during sample processing, and, rather than normalizing to the total number of mapped viral reads in a sample, mapped reads were normalized to phage Q33 abundance (46). Of note, this method of quantification assumes that phages in a sample have a genome size similar to that of Q33, meaning some viruses will be incorrectly estimated (46).

## VIRUS-BACTERIAL HOST DYNAMICS AND VIRAL LIFESTYLE

Once a phage-bacterial host pair of interest is identified, several methods can be employed to characterize their dynamics, ranging from classical virological protocols to high-throughput screens. Below, we will discuss various *in vitro* and *in silico* techniques to examine viral characteristics and virus-bacterial host dynamics.

### *In vitro* methods for examining phage-bacterial interactions

Measuring changes in bacterial optical density (e.g., using a spectrophotometer) over time during incubation with phages is a popular method for characterizing the bacterial host response to phage infection and inferring phage-bacterial host dynamics (173, 174). This technique has been used to characterize phage infection dynamics between gut phages and their bacterial hosts, such as ΦCrAss001 and *Bacteroides intestinalis* (175). Tracking changes in optical density over time can also be used to detect prophage induction, that is, a prophage integrated into the bacterial host genome switching from lysogeny to lytic replication, leading to a drop in bacterial growth (176). Prophage induction is measured in response to an inducing agent such as Mitomycin C. Measuring optical density can serve as a screening tool to study the impact of other stimuli present in the gut environment on phage induction, including antimicrobials (153) and diet-related exposures such as fructose (177) or rebaudioside A (a component of the non-nutritive sweetener stevia) (178). Measuring optical density is simple to execute, fast, and inexpensive. It can therefore be useful as a screening tool, especially when comparing multiple culture conditions (e.g., temperature, MOI, and nutrients) or strains. Notably, this method does not measure bacterial viability and the turbidity is affected by cell debris from lysed and dead cells. The optical density of the culture can also be impacted by bacterial physiology, including changes in cell morphology and biofilm or clump formation in response to infection. Most importantly, this method does not measure phage abundance and does not provide information about viral progeny production or the timing of the viral infection, and it may be unsuitable for detecting infection events that cause minimal changes to optical density, such as temperate infections, abortive infections, or pseudolysogeny (metabolic shutdown without lysis). Therefore, measuring bacterial optical density should be coupled with phage quantification, which can be achieved using plaque assays, microscopy-based techniques, or quantification of phage genetic material (e.g., qPCR).

In line with this need, several assays have been developed to mechanistically interrogate phage-bacterial host interactions and represent robust approaches with high potential to uncover such mechanisms in the gut. Adsorption assays (179) are used to determine the rate of adsorption (or phage attachment), whereas a one-step growth curve (180) is used to quantify phage production over time directly. Both methods were classically used with *E. coli* phages (181), and their applicability to other human gut phages remains to be determined. Furthermore, they rely on plaque formation, making them unsuitable for non-lytic phages. While these assays can yield valuable information on phage-bacterial host interactions, they are limited by their inability to scale up to meet high-throughput demands (182). Thus, high-throughput protocols have been developed to investigate various steps in the viral life cycle. This includes the microscopic phage adsorption assay (MPA), which uses fluorescent microscopy to study phage-bacterial host adsorption at single-virus resolution (182). Notably, MPA is not well-suited for studying phages with small capsid diameters (~25 nm) (182).

Microscopy-based assays can be used to visualize and interrogate phage-bacterial interactions. For example, PhageFISH (183) is an adaptation of fluorescent *in situ* hybridization that labels many target phage genes and the bacterial host rRNA with fluorescent probes. The presence or absence of fluorescence can be used to characterize the phage’s replication cycle and host range (183). Additionally, this technique can also label extracellular phages in the sample. While PhageFISH was initially designed to characterize the phage-bacterial interactions of environmental phages with *Pseudoalteromonas* sp. strain H100 (183), it has more recently been used to confirm the presence of jumbophage assembled from pig fecal metagenomes (184). Notably, probe design for PhageFISH requires knowledge of the target’s genomic sequence.

While phages are the predominant known viral group within the human gut virome, a community of gut commensal eukaryotic viruses that target the mammalian host also exists (76). Commensal eukaryotic viruses in the gut virome exhibit large interindividual variability but commonly include ssDNA viruses belonging to *Anelloviridae*, as well as ssDNA *Circoviridae* and multiple RNA viruses. The techniques used to characterize eukaryotic virus-mammalian host interactions with eukaryotic cells are ones historically used to study pathogenic eukaryotic viruses, but may not be widely applicable to commensal gut viruses. For example, plaque assays can provide insight into a given eukaryotic virus’s host range (157) but are not suitable for quantifying or characterizing viruses that do not readily lyse cells, such as ERVs (152) or viruses difficult to culture *in vitro* like Teno Torque viruses, which belong to *Anelloviridae* and are predicted to be an abundant member of the gut virome (185, 186). RT-qPCR and qPCR are common techniques used to characterize virus-mammalian host interactions for eukaryotic viruses, with a particular focus on how these commensal eukaryotic viruses may alter mammalian host cell metabolism and immune signaling (152, 187, 188). As with the challenge mentioned above for PCR-based assays, the genome of the virus of interest must be known to develop primers for detecting viral genome replication.

### *In silico* phage lifestyle prediction

*In silico* prediction of phage lifestyle can also provide insight into phage-host dynamics. In the gut, a majority of bacteria are predicted to carry a prophage (189, 190). Still, the actual lifestyle makeup of the gut virome has not been fully elucidated, with recent estimates categorizing most gut phages as temperate and integrated into a host genome (191, 192). Prediction of viral lifestyle *in silico* is challenging due to the limited number of reference genomes available and incomplete assembly of viral genomes (106). Tools use training data sets consisting of temperate- or virulence-associated genes, such as integrase and excisionase in temperate viruses, to predict UViG lifestyles (104, 107, 193). However, using this strategy relies on the assumption that a genome is complete. Meaning, if a gene is absent, it is assumed that the gene is truly absent from the genome rather than a result of an incompletely assembled genome. To overcome this, other tools have trained machine learning classifiers on DNA and protein feature data from known temperate and virulent phages to predict the lifestyles of query UViGs. This includes PHACTS (105) and BACPHLIP (104), which are trained for use with complete genomes, and PhaTYP (106) and Deephage (107), which utilize deep learning methods to predict lifestyle. Notably, PhaTYP was designed to handle incomplete UViGs assembled from metagenomes (106). In addition, some viral classification tools, such as VIBRANT and GeNomad, can predict whether a viral genome is integrated as a prophage by identifying regions where bacterial-specific genes flank virus-specific genes (84, 90).

## COMMUNITY DYNAMICS

### Wet lab methods to study virome dynamics

To date, much of the knowledge regarding the role of the gut virome in mammalian and human health is associative in nature, relying on correlations between viral abundances and disease states. While the above *in vitro* approaches provide valuable insights into virome characterization, they cannot reveal the mechanisms by which viruses affect overall mammalian health. Some *in vitro* systems aim to mimic the gut environment, such as the “gut-on-a-chip” model. Interestingly, when examining how phage communities behave in culture, it was found that coevolutionary dynamics between phages and bacterial hosts can be detected in the gut-on-a-chip model but not in standard liquid culture (194), highlighting the importance of culturing conditions when studying phage-bacterial host dynamics *in vitro*. Although the gut-on-a-chip system mimics the intestinal environment more closely than standard culturing conditions, it does not perfectly recapitulate the complexity of the intestinal environment cellular milieu, immune and neuronal interactions, and chemical gradients (195). Additionally, there are instances in which a virus of interest is uncultivable because the bacterial or cellular host for the virus is unknown, unculturable under laboratory conditions (196), or the virus itself cannot produce viable virions reliably under laboratory conditions, as with endogenous retroviruses (152) or Gokushovirus WZ-2015a (9), making them unsuitable for these assays.

*In vivo* mouse models can be used to more comprehensively interrogate gut viruses and their relationship to the gut and mammalian health. The use of FMT in GF mouse models can establish causality and determine the mechanism by which a gut commensal impacts mammalian host health (197). However, FMT can contain any microorganism or bioactive compound from the gut microbiome, making it difficult to determine the direct effect of a given viral population in mediating the observed effects on mammalian host health (198). To better elucidate the direct impact of the virome on mammalian host health, GF mouse models can be mono-colonized with specific gut viruses, which have been shown to simulate the development of the intestinal environment, including intestinal structure and immune populations (23). GF mice can also be colonized with a complex community of gut viruses via FVT, which refers to the enrichment or separation of viruses from other stool-derived cells, metabolites, and debris. FVT preparations rely on enrichment methods discussed earlier (198). Administration of FVT to GF mice has shown that commensal viruses directly interact with the mammalian host immune system or modulate physiology (2, 23) and establish a causal link with IBD pathology (199). FVT has also shown efficacy within non-sterile, specific-pathogen-free (SPF) disease models. This includes a reduction in stress behavior in a mouse model of chronic stress (13) and an improvement of disease parameters in a mouse model of type II diabetes (15). Similar to FVT, the preparation of sterile fecal filtrate transfers (SFFTs) removes most microbes, but not viruses, from the sample. It does not include additional viral enrichment or sample purification steps for cell debris, proteins, or bioactive small molecules such as bile acids (14, 200). In contrast, FVT preparation enriches viral quantity and may include purification steps such as dialysis (13). This allows FVT studies to link viruses to modulation of mammalian host health more conclusively. In the clinic, SFFTs have been used to treat infections such as *C. difficile* (14) and to alleviate symptoms of metabolic syndrome (200).

While a majority of *in vivo* approaches to study the gut virome involve adding viruses, viruses can also be depleted from the host microbiome. One such method involves administering acriflavine (201), a known inhibitor of phage replication. Using this model, it was found that gut phages increase the potency of ampicillin activity toward commensal gut bacteria (201). This recent work provides a foundation for an exciting new approach to mechanistically interrogate the gut virome while leaving other microbial communities undisturbed and requires validation in additional mouse models.

Combining several of the aforementioned techniques can be a powerful approach to simultaneously study the roles of both commensal bacteria and viruses in a phenotype of interest. Experiments comparing antibiotic-treated to antibiotics-naïve animals could be expanded to include groups treated with acriflavine to determine whether host-related readouts are impacted by disruption of bacteria, viruses, or both. Coupling FMT experiments with groups receiving FVT can point to a causal role for viruses in a given phenotype. Finally, contrasting GF and SPF recipients of FVT can help determine whether the viral community impacts the host directly or through modulation of the bacteriome.

### *In silico* prediction of virome function

In addition to determining the viral composition of a sample, *in silico* prediction of genomic features and gene annotation of viral genomes can provide information on the functions performed by a virus or viral community in a given environment and the viral genes involved. For example, phages can confer additional fitness advantages to their bacterial host through auxiliary metabolic genes (AMGs) (22). In the gut, viral AMGs that confer virulence factors have been positively associated with metabolic syndrome in clinical cohorts (6). Notably, viruses have been found to use different genetic codes from those of their bacterial hosts, particularly by using other codons as stop codons (202). Accounting for the use of alternative stop codons in viral genomes significantly increases functional annotation and minimizes misidentification of bacterial genes as viral (203) and has been implemented in widely used genome feature prediction tools, including Prokka (204) and the virus-specific tool, Pharokka (108). Once genes are predicted, they can be annotated with a variety of databases, including comprehensive resources such as Kyoto Encyclopedia of Genes and Genomes (KEGG) (205), Gene Ontology (GO) (206), and Protein Families (Pfam) (207) and virus-specific resources such as the viral orthology group (VOG) database (116) and Prokaryotic Virus Remote Homologous Groups (PHROGS) database (115). Additional tools are being developed to predict protein similarity to known functional proteins based on protein structure rather than sequence similarity, using AlphaFold (208). In addition to classifying sequences, VIBRANT also supplies viral gene prediction and annotation with a “viral score” or “v-score,” which indicates the probability that a given gene is viral. Typically, genes with a v-score >1 are considered viral in origin, while genes with a v-score <1 are considered contamination or bacterial in origin (90).

## VIRAL PIPELINES

To standardize and aid in virome research, pipelines for both *in vitro* and *in silico* methods have been developed. *In silico,* this includes multiple pipelines that include some or all steps discussed above, but vary in specific program usage, which can impact the final predicted viral data set. For example, pipelines vary greatly in the number of viral classification tools employed to predict UViGs from samples. The Modular Viromics Pipeline (209) employs just one classifier, GeNomad (84), whereas Metaphage (210) employs five. Because of the differences in methodologies and biases, it may be tempting to use many tools to improve accuracy and recovery of viral genomes. However, both the number and specific programs used can significantly affect viral contig prediction, in some cases decreasing overall recovery rates (211). Only one pipeline, ViWrap (212), employs genome binning, which increases the completeness of recovered genomes at the cost of some genomic diversity. Some pipelines are fully available on web servers, making them more accessible, such as PhaBOX (213), but online pipelines typically cannot handle large analyses. Additionally, the Modular Viromics Pipeline (209) is designed to be continuously updated with the latest taxonomy and new viromic programs. Finally, programs such as Phanta and Hecatomb do not employ the typical assembly-based workflow as described above (70, 214). Phanta enables the simultaneous profiling of bacteria and viruses by aligning kmers against large bacterial and viral reference databases. Phanta’s assembly-free method has improved characterization of metagenomes sequenced at shallow depth and of short reads (70). Hecatomb attempts to reduce the number of false-positive viral predictions by mapping reads to multiple viral amino acid and nucleotide databases (214). Read annotations are then used to annotate assembled contigs. Hecatomb is also compatible with short and long read sequences. A user’s choice of a viromic pipeline should reflect what best fits their sample. For example, Virsorter2 (122) is well-suited for gut environment samples, while GeNomad can identify a broad range of genome types, as well as other MGEs such as plasmids (84). If samples are sequenced at shallow depth and assembly-based approaches fall short, Phanta uses a read-mapping approach, making it much better suited to that sample type.

*In vitro,* multiple wet lab techniques have been combined to improve the quantity and quality of viral enrichments (49). The “phage on tap” (PoT) protocol, a published workflow for isolating viruses from environmental samples, produces high-titer, low-endotoxin viral stocks in fewer days than classic protocols (49). First, a typical phage plaque assay is performed to determine the starting titer of the phage stock and provide discrete phage plaques. Subsequently, a single plaque is used to propagate the phage by co-culturing it with bacteria in liquid broth. The liquid culture is then centrifuged, ultrafiltered, and chemically separated to purify the amplified phages from bacterial components. The phage stock is then further purified with organic solvents to remove any remaining bacterial endotoxins, leaving a high-titer, homogenous phage stock with minimal bacterial endotoxin contamination. While the major benefit of this pipeline is the high titer and quality of the phage stock, there are still several considerations (49). First, PoT was originally developed with the model T4 phage and *E. coli* (49), which means that further calibration and alterations should be considered when applying PoT to other cultivable phage-host pairs. Another consideration is that PoT is limited to cultivatable hosts. Finally, if the sensitive bacterial host is already a lysogen, the prophage may become active and produce viable virions, resulting in a phage stock that is a mixture of the activated prophage and the phages of interest (215).

## OTHER MGEs

Numerous tools and *in vitro* protocols for extracting and bioinformatically analyzing the gut virome have explicitly been calibrated for use with phages in mind (69). For example, multiple bioinformatic tools have been trained exclusively with phage genomes (82, 83, 98). When studying other MGEs, such as non-pathogenic eukaryotic viruses, plasmids, and integrative and conjugative elements (ICEs), challenges such as limited reference databases and specialized tools are further exacerbated as these entities are even less characterized (216). Additionally, genes contained within MGEs can originate from other mobile elements, adding an additional layer of complexity to prediction (217). Some viral classifiers have been trained on data that includes genomes outside of phages, including eukaryotic viruses (92) and other MGEs (88, 90). In the case of GeNomad, its training set included phage genomes, eukaryotic viral genomes, and other MGEs (84). Including these data sets in classifier training enables tools to detect these entities with high accuracy and sensitivity, as shown in GeNomad’s own benchmarking (84). In the case of ICEs, they can be identified through conserved genes (218) or through the use of developed tools such as ICEfinder (219) and MGEfinder (220) and databases like PlasmidScope (221) and ICEberg (222). When human fecal metagenomes were investigated, it was found that the newly identified pBI143 plasmid was present in ~70% of fecal metagenomes examined and was over 10-fold more abundant than the most abundant phage family, crAssphage (127). The authors then developed PlasX, a machine-learning tool that identifies plasmids in metagenomes. This tool was used to identify 68,000 nonredundant plasmids across human gut metagenomes, highlighting the extreme diversity of underexplored plasmids (216). Given the importance of plasmids to bacterial physiology and, more broadly, to public health through their role in conferring antibiotic resistance and major bacterial virulence factors (223), further development of tools and databases to classify and characterize plasmids is warranted.

## CONCLUDING REMARKS

The landscape of wet-lab experimentation and, even more so, computational tools for studying the human gut virome has considerably broadened in recent years, providing exciting new means of understanding the impact of endogenous viruses on mammalian host physiology. We expect that the growing interest in the human virome will lead to improved methodologies that are both more accessible and address many of the caveats and considerations highlighted in this review. Focusing on improving bacterial host and functional annotation, as well as viral protein databases, will significantly aid the use of computational approaches to understand how the gut virome impacts host physiology. These methods can be further improved by cultivating viruses from a more diverse range of hosts and by sampling the GI tract beyond feces, where even more undiscovered diversity may lie (57). As tools for virome research become more accessible, we hope that researchers can continue to synergize dry lab (Table 1 and Table 2) and wet lab (Table 3) in their study designs. While some of the caveats discussed in this review for individual methods are technical and may be overcome in the future, others are inherently biological and therefore are always likely to require synergizing several methods to address the biological problem. Collectively, we hope that the methods discussed in this review will assist researchers in propelling the field of gut viromics forward and ultimately unlock the potential of the gut virome as a diagnostic marker, therapeutic target, or even a therapy itself through FVT or viral probiotics.

**TABLE 3:** *In vitro* approaches (in alphabetical order) to enriching, isolating, quantifying, and characterizing phages^*a*^

Technique (reference)	Function	Advantages	Disadvantages
AdsorpSeq (65)	Enrichment Phage-bacterium interactions	High throughput With sequencing approaches, can identify phage-bacterial taxonomy	Unable to differentiate viable from unviable phages Culture conditions can affect phage adsorption
Adsorption assay (179)	Phage-bacterium Interactions	Provides information on adsorption rate of phage for a given host	Low throughput Unable to be used on uncultivable phages Biased towards phages that produce visible plaques Culture conditions can affect phage adsorption
Cryogenic transmission electron microscopy (163)	Quantification Visualization/identification	Provides visualization of virion particles Able to visualize unculturable viruses (ex: crAssphages) Culture independent	Cannot distinguish between viable versus non-viable virus
ddPCR (146)	Identification Phage-bacterium interactions	Ability to multiplex several gene targets Ability to characterize phage-host interactions Ability to characterize phage-phage competition for host Culture independent	Genome for the phage of interest is needed for probe design Unable to differentiate viable from unviable phages
epicPCR (149)	Phage identification Phage-bacterium interactions	High throughput and cost-efficient No special equipment required Culture independent	Requires primer design for a phage marker, requires some level of prior knowledge of phage genome
Gut-on-a-chip (194, 195)	Phage-bacterium interactions Phage-mammalian host interactions	Currently, the best model that closely mimics conditions within the GI tract Can sustain mammalian cells, mammalian cell differentiation, and the gut microbiome Can mimic peristalsis Mammalian cells can differentiate into various cell types associated with the GI tract Mammalian cells can secrete mucus	Requires specific, specialized set-up that can be costly Still currently struggles to incorporate the full complexity of the GI immune system and nervous system Does not take into consideration the heterogeneity in the GI environment along the GI tract
Microscopic phage adsorption assay (182)	Phage-bacterium interactions	High throughput Can provide down to a single phage-bacterium interaction resolution Uses a non-specific dye for labeling phages	Cannot accurately track phages <25 nm in diameter due to low fluorescent signal Needs careful controls to ensure that the non-specific dye does not interfere with phage adsorption
Onestep growth curve (180)	Phage-bacterium interactions	Provides information on length of phage replication cycle and burst size	Low throughput Resource intensive Unable to be used on uncultivable phages Biased towards phages that produce visible plaques Culture conditions can impact the phage replication cycle and burst size
PhageFISH (183)	Identification Quantification Phage-bacterium interaction Lifecycle	Provides resolution to single cell level Can track intracellular and extracellular phages Phage and bacterial host specificity	Genome for the phage of interest is needed for probe design Current protocol is incompatible with live imaging Low throughput Culture condition can influence phage adsorption
Phage on tap (49)	Phage amplification Enrichment	Can produce high-titer phage stocks (10^10–11^ PFU/mL) Estimated 3,000-fold decrease in bacterial endotoxin contamination	Only applicable to phages with cultivatable hosts Requires calibration for each phage of interest
Spectrophotometer-based growth curves (173)	Phage-bacterium interactions	Provides an overview of how bacteria behave in response to phage Able to test effects of environmental stimuli on phage- bacterium interactions High-throughput Low-cost	OD measurements are not always indicative of CFU Indirect method of visualizing phage replication Culture conditions are not representative in the context of the gut environment
Viral tagging (62)	Isolation	High-throughput Uses non-specific fluorescent dyes that bind to nucleic acids Culture-independent	Requires a flow cytometer Unable to differentiate viable from nonviable phages Fluorescent dyes could impact phage adsorption
Viral tag and grow (66)	Enrichment Phage-bacterium interactions	High-throughput Uses non-specific fluorescent dyes that bind to nucleic acids Able to differentiate viable from nonviable phages	Requires a flow cytometer Requires a culturable bacterial host Requires further purification since isolated phages are likely heterogeneous Bias toward lytic phages Fluorescent dyes could impact phage adsorption or impact phage viability

^*a*^ Research rigor can be improved through using a combination of these *in vitro* approaches, which is advised as a means to address the advantages and disadvantages of each other.
